# Comparison of Anticancer Activity and HPLC-DAD Determination of Selected Isoquinoline Alkaloids from *Thalictrum foetidum*, *Berberis* sp. and *Chelidonium majus* Extracts

**DOI:** 10.3390/molecules24193417

**Published:** 2019-09-20

**Authors:** Anna Petruczynik, Tomasz Tuzimski, Tomasz Plech, Justyna Misiurek, Karolina Szalast, Grażyna Szymczak

**Affiliations:** 1 Department of Inorganic Chemistry, Medical University of Lublin, Chodźki 4a, 20-093 Lublin, Poland; justyna.misiurek@umlub.pl; 2 Department of Physical Chemistry, Medical University of Lublin, Chodźki 4a, 20-093 Lublin, Poland; 3 Department of Pharmacology, Medical University of Lublin, Chodźki 4a, 20-093 Lublin, Poland; tomasz.plech@umlub.pl (T.P.); karolina.szalast@umlub.pl (K.S.); 4 Botanical Garden of Maria Curie-Skłodowska University in Lublin, 20-819 Lublin, Poland; grazyna.szymczak@poczta.umcs.lublin.pl

**Keywords:** isoquinoline alkaloids, HPLC-DAD, *Chelidonium majus*, *Berberis* sp., *Thalictrum foetidum*, cytotoxic activity, etoposide

## Abstract

*Background:* Plants are an important origin of natural substances that the raw material for various pharmaceutical and therapeutic applications due to the presence of phytochemicals, such as alkaloids. Alkaloids, which are found in different plant species, possess numerous biological activities. Some alkaloids have strong cytotoxic effects on various cancer cells. The search for new drugs to treat various cancers is one of the most important challenges of modern scientific research. *Objective:* This study aimed to investigate of cytotoxic activity of extracts that were obtained from *Chelidonium Majus*; *Berberis* sp.; *Thalictrum foetidum* containing various alkaloids on selected cancer cell lines. The aim was also the quantification of selected alkaloids in the investigated extracts by HPLC. *Methods:* The analysis of alkaloids contents were performed while using HPLC in reversed phase (RP) mode using Polar RP column and mobile phase containing acetonitrile, water, and ionic liquid. The cytotoxic effect of the tested plant extracts and respective alkaloids’ standards were examined while using human pharyngeal squamous carcinoma cells (FaDu), human tongue squamous carcinoma cells (SCC-25), human breast adenocarcinoma cell line (MCF-7), and human triple-negative breast adenocarcinoma cell line (MDA-MB-231). *Conclusion:* All of the investigated plant extracts possess cytotoxic activity against cancer cell lines: FaDu, SCC-25, MCF-7, and MDA-MB-231. The highest cytotoxic activity against FaDu and MDA-MB-231 cells was observed for *Chelidonium majus* root extract, while the highest cytotoxic activity against SCC-25 and MCF-7 cells was estimated for the *Thalictrum foetidum* root extract. There obtained significant differences in the cytotoxic activity of extracts that were obtained from the roots and herbs of *Chelidonium majus* and *Thalictrum foetidum.* Based on these results, investigated plant extracts can be recommended for further investigations of anticancer activity.

## 1. Introduction

Chemotherapy is the treatment of choice in many malignant tumors. Chemotherapy drugs can potentially eliminate cancer cells, but can also damage perfectly healthy cells and tissues; these induce side effects throughout the body, which dramatically reduces the quality of life for patients. Additionally, cancer cells might develop a multidrug resistant phenotype in prolonged treatment [1]. Searching for antitumor agents is the one of the most intensive segment in the development of new drugs. A modern approach to there-search is focused on finding compounds that affect important processes in tumor development, progression, and metastasis. Recently, many investigations have been made for discovering new drugs, including drug from natural sources in order to circumvent drug resistance. Plant-based cancer therapeutics are considered a preferable treatment, as they are natural, easily available, and can be readily used via a dietary intake. Based on past major successes, medicinal plants are considered to be a promising source to develop new chemical compounds with potential anticancer activities.

A large number of plant metabolites are utilized against various diseases, including cancer [2,3]. The herbal medicines have played an important role in the prevention and treatment of cancer, which execute their multiple therapeutic effects by inhibiting cancer activating enzymes and hormones, promoting the production of protective enzymes, inducing antioxidant action and enhancing immunity, stimulating DNA repair mechanism, thus showing an anticancer effect. Several plant products, such as alkaloids, flavonoids, lignans, saponins, terpenes, taxanes, vitamins, minerals, glycosides, gums, oils, biomolecules, and some other primary and secondary metabolites play significant roles in anticancer activity. A strategy to identify safe compounds with anticancer effects is urgently needed to treat various cancers. Alkaloids are a rich as well as an important source for searching for pharmacologically active drugs in anticancer treatment.

Different plant extracts containing various alkaloids were examined on in vitro anticancer activity e.g., *Peganum harmala* containing harmine derivative alkaloids [4], *Pyrenacantha volubilis* containing quinoline indole alkaloid camptothecine [5], *Anthocephalus cadamba* containing dihydrocadambine [6], *Enicosanthellum pulchrum* with cleistopholine [7], *Ophiorrhiza mungos* L. *var. angustifolia* containing anticancer alkaloid camptothecin [8], *Ophiorrhiza rugosa var. decumbens* with camptothecin [9], *Catharanthus roseus* containing anticancer alkaloids vincristine and vinblastine [10,11], and *Stemona aphylla* containing stemocurtisine and oxystemokerrine [12].

The major secondary metabolites are benzylisoquinoline alkaloids, such as chelidonine, sanguinarine, chelerythrine, berberine, and coptisine. Antitumor activity of this plant have been studied in many in vitro and in vivo experiments [13,14]. Most in vitro studies shows that sanguinarine, chelidonine, chelerythrine and berberine are responsible for the antitumoural effect of the *Chelidonium majus* extract. Most previous studies suggested that the strongest anticancer properties are sanguinarine, which intercalates strongly with DNA.

In traditional medicine, extracts of various plants from the *Berberidaceae* family have pharmacological activities, such as in amoebiasis, cholera and diarrhea, possess analgesic and antipyretic effects, antiarrhythmic, anticancer and they used for rheumatic complaints and other types of chronic inflammation [15]. The active constituents of *Berberis* species are alkaloids and the major compound is berberine. Berberine has been used for the treat of variety of cancers, namely breast, prostate, and colorectal cancer [16]. Berberine induces apoptosis in breast, colorectal, and liver cancer, inhibit anti-apoptotic proteins and activate pro-apoptotic proteins.

The genus *Thalictrum* has great medicinal potential due to antiperiodic, diuretic, febrifuge, purgative activity, and anticancer properties [17]. Plants belonging to the genus contained alkaloids, such as: berberine, columbamine, hernandezine, tetrandrine, jatrorrhizine, thalifendine, palmatine, thalidasine, dehydrodiscretamine, tembetarine, xanthoplanine, and magnoflorine.

Some isoquinoline alkaloids exhibit various activities, such as anti-inflammatory, anticancer, antihypertensive, and antibacterial [18]. Isoquinoline alkaloids were often determined in various plant extracts by the HPLC method. Most often, analysis were performed on C18 columns with mobile phases contained organic modifier, water, and most often addition of acids e.g., formic [19,20,21,22,23,24], rarely addition of salts, e.g., ammonium formate [25], buffer at acidic pH [26], and amines, such as trimethylamine [27,28].

In this paper, a HPLC method using a phenyl column and mobile phase containing the addition of ionic liquid for the separation of some isoqinoline alkaloids in *Chelidonium majus*, *Berberis vulgaris, Berberis thunbergii,* and *Thalictrum foetidum* were described. We also evaluate the cytotoxic effects of plant extracts containing isoquinoline alkaloids on various cancer cell lines.

## 2. Results and Discussion

### 2.1. Optimization of HPLC-DAD

Alkaloid standards (see Table 1) were chromatographed on Polar RP 8 column in eluent system containing 28% acetonitrile, water, and 0.04 ML^−1^ of 1-butyl-3-methylimidazolium tetrafluoroborate. The chromatographic condition was based on the previously published method applied for the determination of isoquinoline alkaloids [28] after appropriate modification. Retention times for investigated alkaloid standards are presented in Table 1 chromatograms as supplementary material (Figure S1A–I) The quantitative analysis was performed by the calibration curve method. Calibration curves were constructed by analyzing the samples at eight concentrations, ranging from 0.001 to 0.2 mg/mL. All of the calibration curves were linear over the concentration ranges with correlation coefficients (r) higher than 0.9990. The limit of detection (LOD) and limit of quantification (LOQ) obtained for alkaloids are presented in Table 1. LOD and LOQ were calculated according to the formula: LOD = 3.3 (SD/S) and LOQ = 10 (SD/S), where SD is the standard deviation of response (peak area) and S is the slope of the calibration curve.

**Figure 1:**
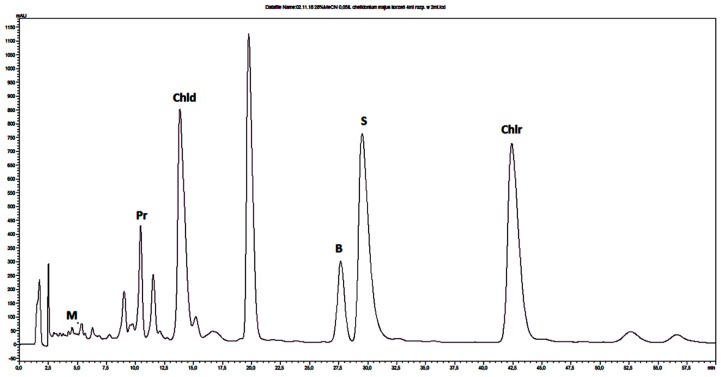
Chromatogram obtained for *Chelidonium majus* root extract obtained on Polar RP column with mobile phase containing 28% MeCN, water and 0.04 ML^−1^ IL. Abbreviations: M-magnoflorine, Pr-protopine, Chld-chelidonine, B-berberine, S-sanquinarine, Chlr-chelerythrine.

**Figure 2:**
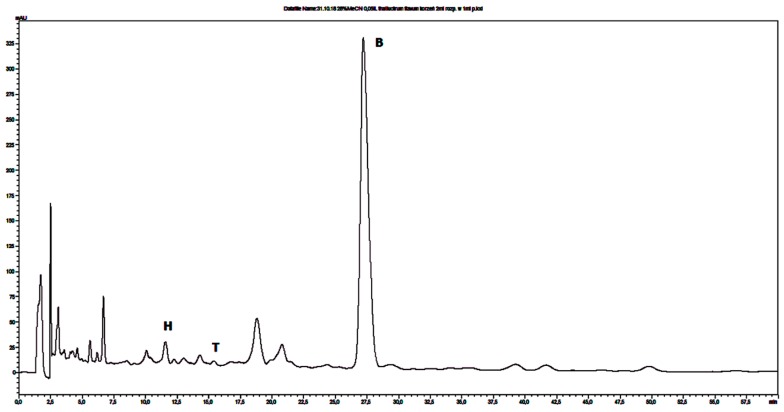
Chromatogram obtained for *Thalictrum foetidum* root extract obtained on Polar RP column with mobile phase containing 28% MeCN, water and 0.04 ML^−1^ IL. Abbreviations: H-hernandezine, T-tetrandrine, B-berberine.

The same chromatographic conditions was developed for quantitative determination of selected isoquinoline alkaloids in plant extracts. The identities of the analyte peaks in plant extracts were confirmed by the comparison of their retention times and UV spectra with the retention times and the spectra of alkaloid standards. Isoquinoline alkaloids were quantified in extracts that were obtained from herb or root of *Chelidonium majus* and *Thalictrum foetidum* and from cortex of *Berberis vulgaris* and *Berberis thunbergii*. Examples of obtained chromatograms are presented in Figure 1 and Figure 2 and supplementary material (Figures S2–S5).

Great differences in the contents of investigated alkaloids have been observed not only in various plant species, and also in herb and root of the same plant species and between the extracts obtained from two *Berberis* spcies (Table 2). The highest content of isoquinoline alkaloids were determined in extract that was obtained from root of *Chelidonium majus;* the lowest in the extract from herb of *Chelidonium majus.* Berberine was determined in all investigated extracts. The highest content of the alkaloid was in extract that was obtained from bark of *Berberis thunbergii* (above 0.6 mg/g of plant material), high content of the alkaloid was also determined in the extract from cortex of *Berberis vulgaris* (above 0.5 mg/g of plant material), and also in extract that was obtained from *Thalictrum foetidum* root (more than 0.3 mg/g). The presence of palmatine was found in extracts obtained from *Berberis* sp. A much higher content of this alkaloid was observed in *Berberis vulgaris* cortex extract. The highest content of chelidonine was determined in extract from root of *Chelidonium majus*—more than 0.95 mg/g of plant material. In the same extract, high concentrations of sanquinarine were detected (above 0.4 mg/g. Great difference in the content of chelerythrine was observed between extracts that were obtained from herb and root of *Chelidonium majus.* Only 0.0026 mg/g of plant material was determined in extract that was obtained from herb while more than 0.23 mg of chelerythrine in g of the plant material was quantified in extract that was obtained from root of the plant. The lowest differences in the content of protopine in herb and root *Chelidonium majus* extracts were observed (0.0975 and 0.1610 mg/g, respectively). Hernandezine and tetrandrine were detected in *Thalictrum foetidum* herb and root extracts. A very high content of hernandezine was found in the root of *Thalictrum foetidum* (more than 1 mg/g of plant material), while only 0.0377 mg/g was determined in the herb of the plant. In *Thalictrum foetidum* herb, a higher content of tetrandrine was detected (0.7873 mg/g of plant material) when compared to the content of the alkaloid in *Thalictrum foetidum* root (0.5644 mg/g of plant material).

### 2.2. Investigation of In Vitro Anticancer Activity of Alkaloid Standards

The cytotoxic activity of alkaloid standards: berberine, chelidonine, chelerythrine, hernandezins, magnoflorine, palmatine, protopine, sanquinarine, and tetrandrine were carried out while using following human cancer cell lines: human pharyngeal squamous carcinoma cells (FaDu), human tongue squamous carcinoma cells (SCC-25), human breast adenocarcinoma cell line (MCF-7), and human triple-negative breast adenocarcinoma cell line (MDA-MB-231). The results were expressed as IC_50_ values, which represent the concentrations that are required for 50% inhibition of cells viability (Table 3).

For comparison of cytotoxic activity, experiments were also performed for etoposide on the same cell lines and at the same concentrations as the alkaloid standards. Investigated isoquinoline alkaloids had various cytotoxic effects on the examined carcinoma cells.

The highest cytotoxic properties were observed for chelerythrine, which exhibit a very high cytotoxic effect on all applied cell lines. At concentration 10 µg/mL of chelerythrine, the viability of cells was below 10%, at concentration 100 µg/mL decreased to about 2 or less percent. The strongest cytotoxic effect was observed for the FaDu cell line. Similarly, very high cytotoxic activity was found for sanquinarine. At the lowest concentration, viability of all cells was below 10%, and at the highest was about 2.5 or less percent. High activity was also obtained for berberine, especially against SCC-25, FaDu, and MDA-MB-231 (viabilities of cells were 2.78, 13.19, and 14.81%, respectively, for concentration 100 µg/mL). High cytotoxic activity was also determined for tetrandrine, especially against SCC-25, MDA-MB-231, and MCF-7 cell lines (viability of cells were below 0.2% at concentration 100 µg/mL). Additionally, for sanquinarine, the highest cytotoxicity was found against the FaDu cell line. Lower cytotoxicity against most cell lines was determined for magnoflorine, chelidonine, protopine, and palmatine.

### 2.3. Investigation of In Vitro Anticancer Activity of Plant Extracts

**Figure 3:**
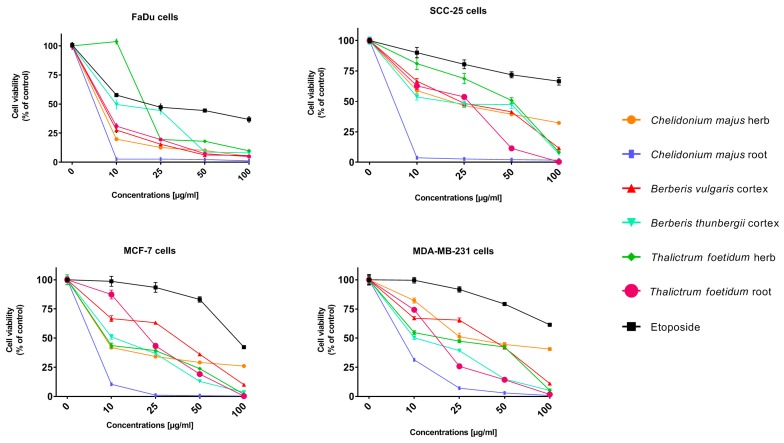
Dependence of human cancer cell viability on concentrations of plant extracts and etoposide.

Afterwards, experiments on the in vitro anticancer activity of extracts that were obtained from herb or root of *Chelidonium majus* and *Thalictrum foetidum*, and from cortex of *Berberis vulgaris* and *Berberis thunbergii* were carried out while using the same cancer cell lines as applied in investigations of cytotoxic activity of alkaloid standards. Preliminary evaluation of cytotoxic properties of the investigated plant extracts was performed while using the following concentrations: 10, 25, 50, and 100 µg/mL. Further testing directed at assessing the IC_50_ values for the plant extracts were carried out due to promising research findings. The extracts were assayed in at least eight different concentrations in order to calculate the respective IC_50_ from the logarithmic dose-response curve (Table 4). Etoposide, anticancer drug was used as a reference substance. The results were reported as the percent growth of the treated cells when compared to the untreated control cells (Figure 3) and IC_50_ (Table 4). For almost all cases (except obtained for *Thalictrum foetidum* herb extract at concentration 10 µg/mL), lower cell viability was observed after treatment by all investigated plant extracts at all tested concentrations as compared to etoposide. The obtained results indicate very high cytotoxic activity of all investigated extracts.

The greatest cytotoxic properties were observed in cells that were treated by *Chelidonium majus* root extract. The results showed that when the extract concentration added to the cell culture was increased, all the tested cell viability decreased (Figure 3). After application of the extract in a concentration of only 10 µg/mL, FaDu and SCC-25 cells viability were only 2.68 and 3.67%, respectively. At the same concentration of etoposide, viability of the same cell lines were 57.7% and 90.2%, respectively.

Cells viability additionally decreased with the increase of extract concentrations; and at concentration of 100 µg/mL was lower than 2% for all treated cells. The values of the cells viability of MCF-7 and MDA-MB-231 were only 0.47% and 0.84%, respectively. The viability of MCF-7 and MDA-MB-231 cells that were treated by etoposide at the same concentration were 86.39% and 79.22%, respectively.

The cytotoxicity of *Chelidonium majus* herb extract was also evaluated and compared with activity of extract obtained from root of the same plant. The herb extract demonstrates low reduction of cell viability compared to root extract, but still the reduction is higher than 50% for all cell lines when cells were treated by the extract at the concentration of 100 µg/mL. The activity was also higher than etoposide in all concentrations against all tested cancer cell lines. For the extract, a greater variation in its cytotoxic activity on various cell lines has been observed. The highest cytotoxic effect of herb extract was observed for the FaDu cell line. The viability of cells that were treated by the herb extract at concentration 10 µg/mL was 19.81% and at concentration of 100 µg/mL was only about 5%. Cancer cells SCC-25, MCF-7, and MDA-MB-231 treated by *Chelidonium majus* herb extract at concentration of 10 µg/mL viability were about 59, 35, 52, and 42%, respectively, while after the addition of herb extract at concentration of 100 µg/mL decreased to 32, 31, 41, and 32%, respectively.

The *Chelidonium majus* root has higher all determined alkaloid contents as compared to extract that is obtained from herb. The highest contents of chelerythrine, sanquinarine, and berberine possessing very high cytotoxic activity were found in the root extract. These isoquinoline alkaloids might have synergistic high cytotoxic activity on cancer cell lines.

Extracts that were obtained from *Chelidonium majus* exhibit very strong cytotoxicity. Previous investigations indicated the strong cytotoxicity of these extracts against PANC-1 (IC_50_, 20.7 μg/mL) and HT-29 (IC_50_, 20.6 μg/mL), and a moderate cytotoxic activity against MDA-MB-231(IC_50_, 73.9 μg/mL) [14], A-549, HeLa, and SGC-7901 cell lines [15]. Our studies have shown that the extracts from Chelidonium majus are also cytotoxic against FaDu, SCC-25, MCF-7, MDA-MB-231, and CRL1634 cell lines. Values of IC_50_ that were obtained by us are presented in Table 4. FaDu and SCC-25 cell lines belong to so-called head and neck squamous cell carcinomas are often resistant to chemotherapy, even including targeted drug therapy.

High cytotoxicity in the investigations on the same cell lines was also obtained for *Berberis vulgaris* cortex and *Berberis thunbergii* cortex extracts. Obtained results indicate that the two *Berberis* species cortex extracts were cytotoxic against all tested cell lines, although its cytotoxicity was different against various cells and it strongly depended on the extract concentration.

The lowest cell viability after treating by *Berberis vulgaris* cortex extract at concentration 10 µg/mL was obtained for the FaDu cell line (27.7%). After the addition of the extract at the highest tested concentration (100 µg/mL), the viability of all cells decreased, while highest for SCC-25 and MDA-MB-231 (about 11%). Extract that was obtained from *Berberis thunbergii* cortex exhibited the higher cytoxicity when compared to the *Berberis vulgaris* cortex extract against SCC-25, MCF-7, and MDA-MB-231 cell lines, while cytotoxicity was lower against the FaDu cell line. The viability of all cell lines by the addition of etoposide was significant lower than viability of the same cell lines treated by *Berberis* sp. extracts. In *Berberis thunbergii* extract higher content of berberine possessing high cytotoxic activity against most tested cancer cell lines was determined and for this reason its in vitro anticancer activity was higher than *Berberis vulgaris* extract against most cancer cell lines. The higher cytotoxic activity of *Berberis vulgaris* cortex extract against FaDu cell line might be caused by higher content of palmatine with exhibited high cytotoxicity on the cell line.

Our investigations confirm the previously described cytotoxic activity of the *Berberis vulgaris* extract against MCF-7 cells [29,30] and exhibit the cytotoxic activity against FaDu, SCC-25, and MDA-MB-231 cells. IC_50_ for tested cancer cells were between 5.05 μg/mL for FaDu cells to 35.93 μg/mL for MDA-MB-231 cells. Very strong cytotoxic activity of *Berberis thunbergii* extract was also found against all cancer cells (IC_50_ from 11.57 μg/mL against FaDu to 12.51 μg/mL against MDA-MB-231 cells) (Table 4). The cytotoxic activity of extracts obtained from cortex of *Berberis thunbergii* has not been investigated previously.

The viability of the same cell lines were investigated to examine of *Thalictrum foetidum* herb and root extracts cytotoxity. These extracts exhibit a high cytotoxic properties against all treated cell lines, especially at higher concentrations. Significant differences in the cytotoxicity of both extracts were observed. When extracts at a concentration of 10 μg/mL were applied, the toxicity of both extracts was not too high and a greater cytotoxicity of the extract from herb was observed in relation to the three cell lines. An increase in the concentration of extracts resulted in a very significant reduction in cell viability. Particularly low viability of cells was observed after treatment by the extracts that were obtained from the *Thalictrum foetidum* at a concentration of 100 μg/mL (in all cases viability was lower than 10%). At higher concentrations, the extract that was obtained from the root of *Thalictrum foetidum* as compared to the extract obtained from herb of the plant showed higher cytotoxicity in relation to all cell lines. Especially, high cytotoxicity of the extract that was obtained from the root was observed in relation to the cancer cell lines SCC-25, MCF-7 (viability lower than 1%). Additionally, *Thalictrum* sp. extracts that were obtained both from herb and especially from root showed a much stronger cytotoxic effect on tested cell lines when compared to etoposide. The significantly higher cytotoxic activity of root extract as compared to the extract obtained from herb might be caused by the significantly higher contents of berberine and hernandezine in the part of *Thalictrum foetidum.* The obtained low values of IC_50_ for *Thalictrum foetidum* extracts also indicated strong cytotoxic activity against all cancer cell lines tested by us. An especially low IC value was obtained for extract from root against FaDU cells (IC_50_ = 4.98 μg/mL), low IC_50_ values were also obtained after the treatment of MCF-7 cells by extract from *Thalictrum foetidum* herb (IC_50_=11.1 μg/mL), MDA-MB-231 cell lines (IC_50_ = 17.8 μg/mL) (Table 4).

## 3. Materials and Methods

### 3.1. Apparatus and HPLC Conditions

Analysis was performed while using an LC-20AD Shimadzu (Shimadzu Corporation, Canby, OR, USA) liquid chromatograph that was equipped with column Synergi polar RP 80A (150 × 4.6 mm, 5 μm). The chromatograph was equipped with a Shimadzu SPD-M20A detector (Shimadzu Corporation, Canby, OR, USA). Detection was carried out at a wavelength of 240 nm. All of the chromatographic measurements were controlled by a CTO-10ASVP thermostat (Shimadzu Corporation, Canby, OR, USA). The eluent flow rate was 1.0 mL/min. The extracts were injected into the columns while using the Rheodyne 20 μL injector. The DAD detector was set in the 200–800 nm range. Data acquisition and processing were carried out with LabSolutions software (Shimadzu Corporation, Kyoto, Japan).

### 3.2. Chemicals and Plant Materials

Acetonitrile (MeCN), methanol (MeOH), and 1-butyl-3-methylimidazolium tetrafluoroborate of chromatographic quality were obtained from E. Merck (Darmstadt, Germany), dimethyl sulfoxide (DMSO) was from Sigma-Aldrich (Saint Louis, MO, USA).

Alkaloid standards (berberine, boldine, chelidonine, and papaverine) were purchased from Sigma-Aldrich (St. Louis, MO, USA). Columbamine, magnoflorine, palmatine, sanguinarine, chelerythrine hernandezine, and tetrandrine, were purchased from Chem Faces Biochemical Co. Ltd. (Wuhan, China).

Plant material was collected and identified in the Botanical Garden of Maria Curie-Skłodowska University in Lublin (Poland) in spring and summer 2016. *Chelidonium majus* was collected in May of 2017.

The plants were divided into roots and aboveground parts. Plants organs were cut into pieces and dried at ambient temperature for 1–2 weeks. Branches of *B. vulgaris* were decorticate and dried for 1–2 weeks under the same conditions.

### 3.3. Extraction Procedure

The weighted samples (5 g) of each plant were macerated with 100 mL ethanol for 72 h and continuously extracted in an ultrasonic bath for 5 h. Extracts were filtered, the solvent evaporated under vacuum, and the residues dissolved in 30 mL of 2% sulfuric acid and defatted with diethylether (3 × 40 mL). The aqueous layers were subsequently basified with 25% ammonia to a pH of 9.5–10 and the alkaloids were extracted with chloroform (3 × 50 mL). After evaporation of the organic solvent, the dried extracts were dissolved in 5 mL MeOH prior to HPLC analysis.

### 3.4. Investigation of Cytotoxic Activity

Cytotoxic properties of the tested plant extracts and respective secondary metabolites’ standards were examined while using human pharyngeal squamous carcinoma cells (FaDu), human tongue squamous carcinoma cells (SCC-25), human breast adenocarcinoma cell line (MCF-7), and human triple-negative breast adenocarcinoma cell line (MDA-MB-231). Human normal skin fibroblasts (CRL-1634) and HepG2 cells were used as reference cell lines. FaDu and HepG2 cells were cultured while using Eagle’s Minimum Essential Medium (MEM) supplemented with 10% fetal bovine serum, 100 U/mL of penicillin, and 100 mg/mL of streptomycin. SCC-25 was cultured in Dulbecco’s Modified Eagle’s Medium/Nutrient Mixture F-12 Ham (DMEM/F12) supplemented with 10% fetal bovine serum, 400 ng/mL hydrocortisone, 100 U/mL of penicillin, and 100 mg/mL of streptomycin (all from Sigma Aldrich). MCF-7, MDA-MB-231, and CRL-1634 cells were cultured while using Dulbecco’s Modified Eagle’s Medium—high glucose (DMEM) containing 10% fetal bovine serum, 100 U/mL of penicillin and 100 mg/mL of streptomycin. The cells were maintained at 37 °C in a 5% CO_2_ atmosphere. The dried plant extracts and standards were both dissolved in DMSO in order to obtain stock solutions at concentrations of 250 mg/mL and 50 mg/mL, respectively. At the day of experiment, the suspension of cells (1 × 10^5^ cells/mL) in respective medium containing 10% FBS was applied to a 96-well plate at 100 μL per well. After 24 h of incubation, the medium was removed from the wells and replaced by different concentrations (10–100 µg/mL) of plant extracts or standards in medium containing 2% FBS. The control cells were only cultured with a medium containing 2% FBS. Cytotoxicity of DMSO was also checked at concentrations present in respective dilutions of stock solutions. After 24 h incubation, 15 μL MTT working solution (5 mg/mL in PBS) was added to each well. The plate was incubated for 3 h. Subsequently, 100 μL of 10% SDS solution was added to each well. Cells were incubated overnight at 37 °C to dissolve the precipitated formazan crystals. The concentration of the dissolved formazan was evaluated by measuring the absorbance at λ = 570 nm while using a microplate reader (Epoch, BioTek Instruments, Inc., Winooski, VT, USA). Two independent experiments were performed in triplicate. The viability of cells that were incubated with plant extract or standards was expressed as % of the viability of control (untreated) cells (Figure 3). The investigated standards and extracts were subsequently assayed in at least eight different concentrations in order to calculate the respective IC_50_ from the logarithmic dose-response curve (Table 3 and Table 4). The results of the MTT assay were expressed as mean ± SD. DMSO in the concentrations present in the dilutions of stock solutions did not influence the viability of the tested cells.

## 4. Conclusions

It is evident from the present study that investigated plant extracts are promising and effective research area. The cytotoxic activity of all the investigated plant extracts at almost all concentrations against tested cell lines was significantly higher than activity of anticancer drug etoposide.

All of the tested plant extracts contained isoquinoline alkaloids with high cytotoxic activity. In the root of *Chelidonium majus* a large amount of chelidonine (above 0.95 mg/g of plant material), sanquinarine (above 0.40 mg/g of plant material), and cheleythrine (above 0.23 mg/g of plant material) were determined. Extracts that were obtained from cortex of *Berberis vulgaris* and *Berberis thunbergii* contained large amounts of berberine (0.5106 and 0.6181 mg/g of plant material).

The highest activity against all tested cancer cell lines was found for chelerithrine, sanguinarine, tetrandrine, and berberine. All of the investigated standards of alkaloids had the strongest cytotoxic activity on SCC-25 and MCF-7 cells as compared to etoposide. The weaker cytotoxic activity as compared to etoposide on SCC-25 cells was only magnoflorine. The viability of FaDu cells was lower for five alkaloid standards as compared to the anticancer drug. Most of the alkaloids should be further investigated for in vitro cytotoxic activity and in vivo anticancer studies.

All of the investigated extracts were found to be effective against the cancer cell lines: FaDu, SCC-25, MCF-7, and MDA-MB-231. The highest cytotoxic activity against FaDu and MDA-MB-231cells (viability of cells at concentration of 100 μg/mL were 1.16% and 1.80%, respectively) was observed after applying the *Chelidonium majus* root extract, while the highest cytotoxic activity against SCC-25 and MCF-7 cells (viability of cells at concentration of 100 μg/mL were 0.31% and 1.730%, respectively) was estimated after treating by *Thalictrum foetidum* root extract.

Significant differences were in the cytotoxic activity of extracts that were obtained from the roots and herb of *Chelidonium majus* and *Thalictrum foetidum.* At higher concentrations of plant extracts (50 and 100 µg/mL), the extracts that were obtained from the roots of both plants showed a higher cytotoxic effect. For example, the viability of MDA-MB-231 cells treated by *Chelidonium majus* herb extract was 40.64%, but treated by *Chelidonium majus* root extract was only 0.84%. The differences between the activities of the different parts of the plant strongly depend on their alkaloids composition; also, a synergic effect of the different alkaloids cannot be excluded.

Based on these results, the investigated plant extracts can be recommended for further in vivo experiments. The investigated extracts and alkaloids that are contained therein might be developed as a new candidate anticancer agent for pharyngeal squamous carcinoma, tongue squamous carcinoma, breast adenocarcinoma, and triple-negative breast adenocarcinoma.

## Figures and Tables

**Table 1:** Equation of calibration curve, correlation coefficients (r), limit of detection (LOD) and limit of quantification (LOQ) values.

Alkaloid	t_R_	Equation of Calibration Curve	r	LOD	LOQ
berberine	27.67	y = 72178227x − 370170	0.9993	0.0151	0.0457
chelidonine	13.49	y = 165057880x − 35518	0.9992	0.0011	0.0034
chelerythrine	42.50	y = 84228691x + 413980	0.9998	0.0040	0.0123
hernandezine	11.66	y = 385284475x + 32803	0.9999	0.0004	0.0011
magnoflorine	3.78	y = 23972503x + 263324	0.9992	0.0095	0.0287
palmatine	20.23	y = 51166752x + 511129	0.9994	0.0108	0.0327
protopine	10.34	y = 7344826x + 64160	0.9992	0.0095	0.0288
sanguinarine	29.41	y = 80589787x + 606317	0.9990	0.0123	0.0371
tetrandrine	15.57	y = 341397250x + 61276	0.9999	0.0004	0.0012

**Table 2:** Contents of alkaloids in plant samples and yield of extracts.

Name of Compound.	Content of Alkaloids (mg/g of Plant Material)					
	*Chelidonium majus* Herb	*Chelidonium majus* Root	*Thalictrum foetidum* Herb	*Thalictrum foetidum* Root	*Berberis vulgaris* Cortex	*Berberis thunbergii* Cortex
Berberine	0.0092 ± 0.0012	0.0284 ± 0.0018	0.0007 ± 0.00012	0.3076 ± 0.021	0.5106 ± 0.031	0.6181 0.032
Chelidonine	0.0762 ± 0.0061	0.9558 ± 0.0589	ND	ND	ND	ND
Chelerythrine	0.0026 ± 0.00031	0.2341 ± 0.014	ND	ND	ND	ND
Hernandezine	ND	ND	0.0377 ± 0.0028	1.0665 ± 0.0083	ND	ND
Magnoflorine	ND	0.0275 ± 0.0011	ND	0.0205 ± 0.0014	ND	ND
Palmatine	ND	ND	ND	ND	0.2737 ± 0.019	0.0568 ± 0.0041
Protopine	0.0975 ± 0.0072	0.1610 ± 0.0091	ND	ND	ND	ND
Sanquinarine	0.0104 ± 0.0011	0.4099 ± 0.027	ND	ND	ND	ND
Tetrandrine	ND	ND	0.7873 ± 0.039	0.5644 ± 0.038	ND	ND
**Yield of extract (mg/g of plant material)**						
	16.49	13.58	8.01	7.08	9.32	9.72

ND- not determined.

**Table 3:** Cytotoxic effect of the investigated alkaloids against cancer cell lines (FaDu, SCC-25, MCF-7, MDA-MB-231).

	IC_50_ [µg/mL] ± SD			
	FaDu	SCC-25	MCF-7	MDA-MB-231
Berberine	11.22 ± 2.74	34.36 ± 3.16	46.26 ± 5.99	20.82 ± 3.70
Chelidonine	189.28 ± 17.83	173.63 ± 11.74	85.66 ± 4.82	72.54 ± 8.92
Chelerythrine	2.13 ± 0.11	2.61 ± 0.27	3.17 ± 0.19	2.48 ± 0.09
Hernandezine	113.42 ± 12.43	38.63 ± 2.43	31.71 ± 1.86	18.77 ± 0.38
Magnoflorine	> 200	> 200	> 200	> 200
Palmatine	33.22 ± 3.31	112.85 ± 16.33	160.26 ± 8.64	149.20 ± 12.06
Protopine	83.03 ± 13.27	105.57 ± 11.81	151.80 ± 12.34	108.54 ± 7.84
Sanguinarine	0.28 ± 0.01	0.47 ± 0.04	0.28 ± 0.02	0.42 ± 0.01
Tetrandrine	33.11 ± 4.65	16.60 ± 2.01	4.28 ± 0.82	3.03 ± 0.20

**Table 4:** Cytotoxic effect of the investigated plant extracts against cancer cell lines (FaDu, SCC-25, MCF-7, MDA-MB-231).

Plant Sample	IC_50_ [µg/mL] ± SD			
	FaDu	SCC-25	MCF-7	MDA-MB-231
*Chelidonium majus* herb	4.07 ± 0.77	22.92 ± 4.18	5.85 ± 1.24	27.77 ± 4.45
*Chelidonium majus* root	2.52 ± 0.25	2.68 ± 0.22	1.99 ± 0.09	2.61 ± 0.33
*Berberis vulgaris* cortex	5.05 ± 0.52	27.57 ± 5.68	31.40 ± 5.06	35.93 ± 6.72
*Berberis thunbergii* cortex	11.57 ± 2.03	21.38 ± 4.05	12.27 ± 2.16	12.51 ± 2.08
*Thalictrum foetidum* herb	20.64 ± 3.81	46.03 ± 11.16	11.10 ± 2.17	23.41 ± 3.90
*Thalictrum foetidum* root	4.98 ± 0.60	23.07 ± 4.02	24.60 ± 2.85	17.80 ± 2.72

## Supplementary Materials

The following are available online. Figure S1. Chromatograms obtained for alkaloid standards obtained on Polar RP column with mobile phase containing 28% MeCN, water and 0.04 ML^−1^ IL. (A) berberine, (B) chelerythrine. (C) chelidonine, (D) hernandezine, (E) magnoflorine, (F) palmatine, (G) protopine, (H) sanquinarine, (I) tetrandrine. Figure S2. Chromatogram obtained for Chelidonium majus root extract obtained on Polar RP column with mobile phase containing 28% MeCN, water and 0.04 ML^−1^ IL. Abbreviations: Pr-protopine, Chld-chelidonine, B-berberine, S-sanquinarine, Chlr-chelerythrine. Figure S3. Chromatogram obtained for Berberis vulgaris cortex extract obtained on Polar RP column with mobile phase containing 28% MeCN, water and 0.04 ML^−1^ IL. Abbreviations: P-palmatine, B-berberine. Figure S4. Chromatogram obtained for Berberis thunbergii cortex extract obtained on Polar RP column with mobile phase containing 28% MeCN, water and 0.04 ML^−1^ IL. Abbreviations: P-palmatine, B-berberine. Figure S5. Chromatogram obtained for Thalictrum foetidum herb extract obtained on Polar RP column with mobile phase containing 28% MeCN, water and 0.04 ML^−1^ IL. Abbreviations: H-hernandezine, T-tetrandrine, B-berberine.
