# Supplementary material for: Comparison of Anticancer Activity and HPLC-DAD Determination of Selected Isoquinoline Alkaloids from Thalictrum foetidum, Berberis sp. and Chelidonium majus Extracts
Source: Molecules. 2019 Sep 20;24(19):3417. doi: 10.3390/molecules24193417 (PMC6803846; doi:10.3390/molecules24193417)
Supplement: Supplementary file 1 [file molecules-24-03417-s001.pdf]

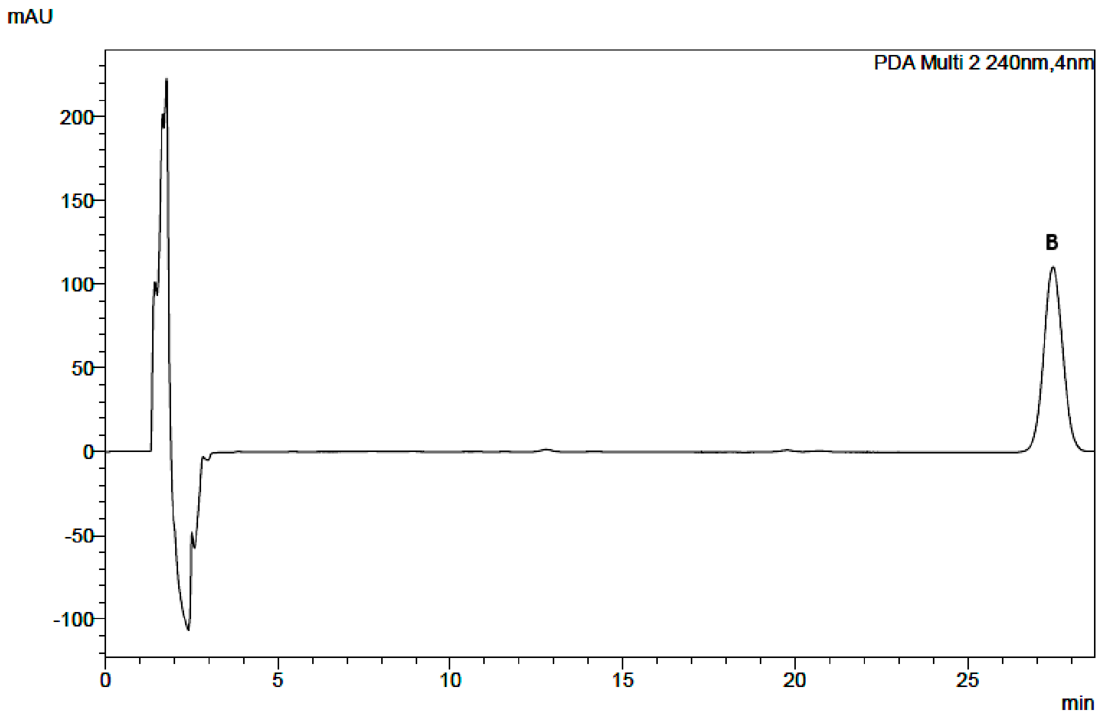

Figure S1A. Chromatogram obtained for berberine on Polar RP column with mobile phase containing 28% MeCN, water and 0.04 ML^{-1} IL.

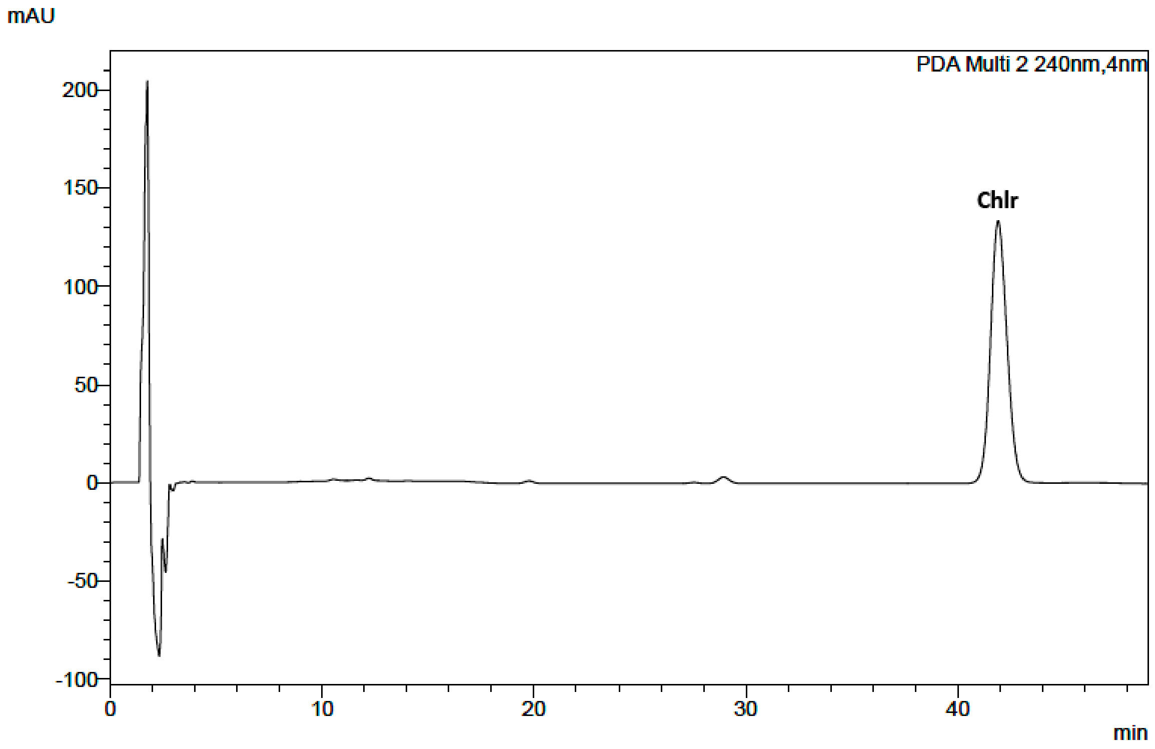

Figure S1B. Chromatogram obtained for chelerythrine on Polar RP column with mobile phase containing 28% MeCN, water and 0.04 ML^{-1} IL.

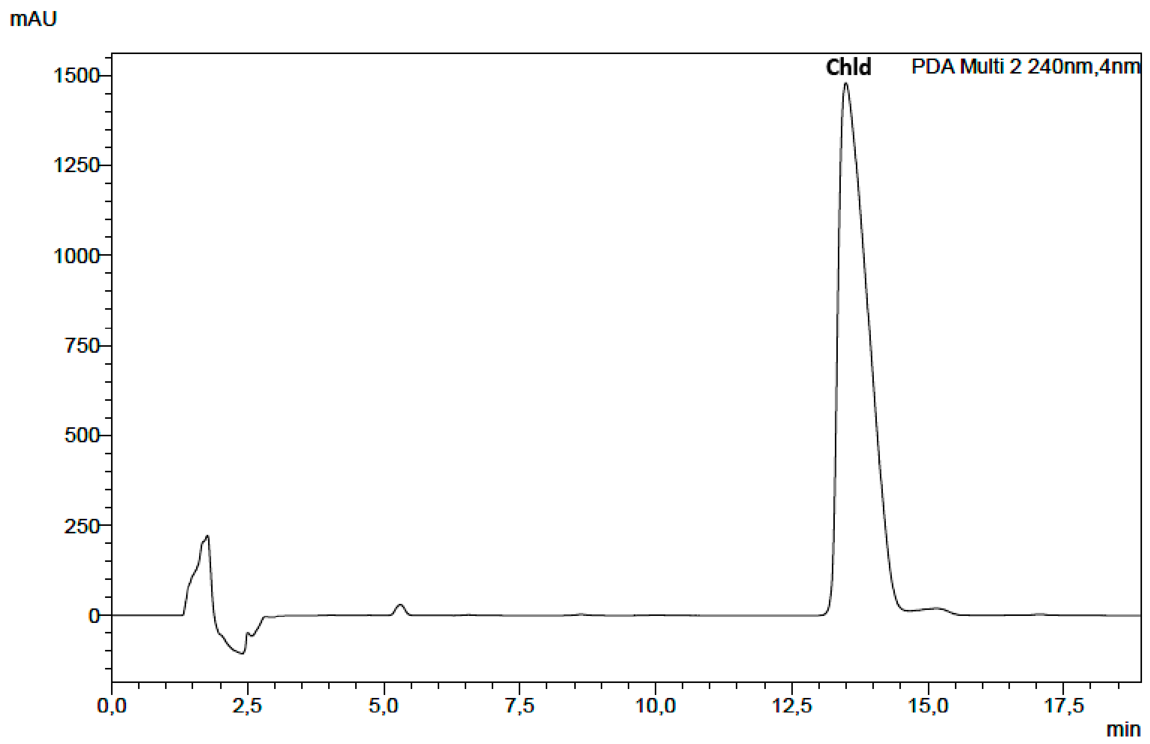

Figure S1C. Chromatogram obtained for chelidonine on Polar RP column with mobile phase containing 28% MeCN, water and 0.04 ML^{-1} IL.

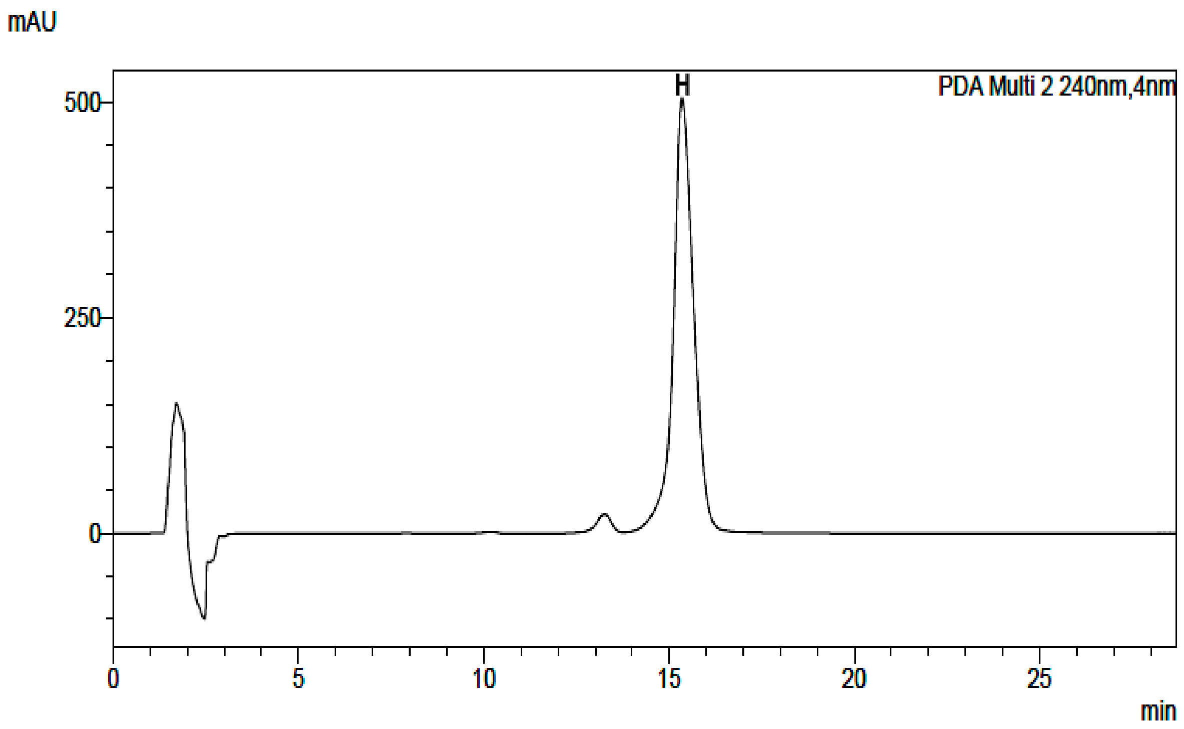

Figure S1D. Chromatogram obtained for hernandezine on Polar RP column with mobile phase containing 28% MeCN, water and 0.04 ML^{-1} IL.

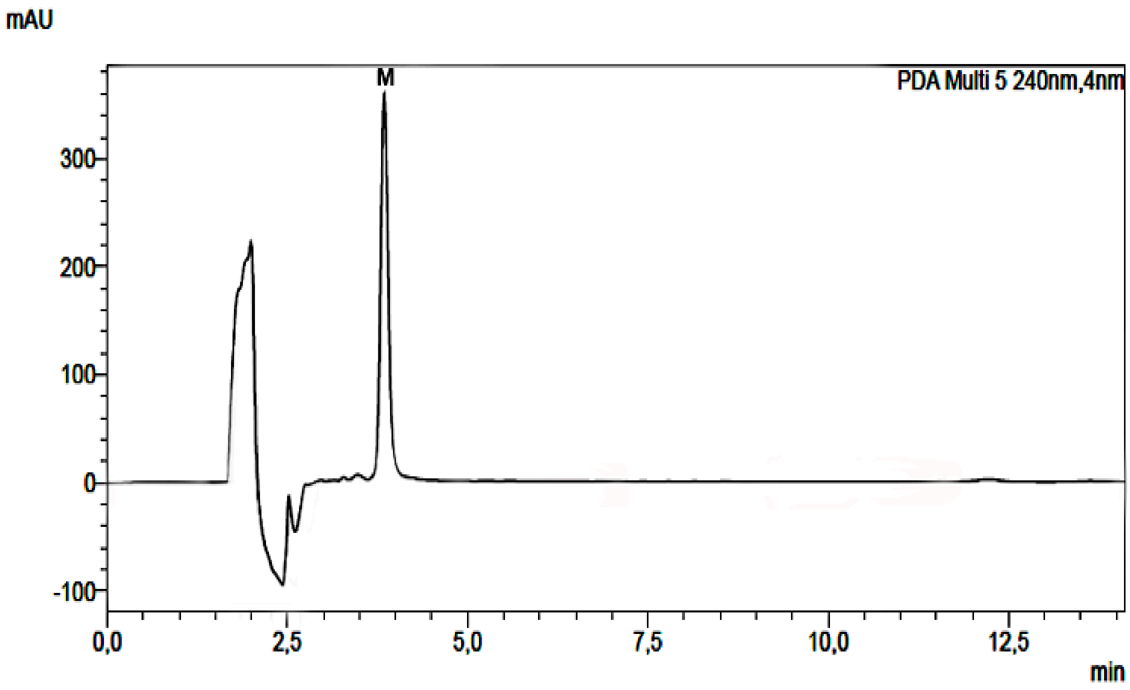

Figure S1E. Chromatogram obtained for magnoflorine on Polar RP column with mobile phase containing 28% MeCN, water and 0.04 ML^{-1} IL.

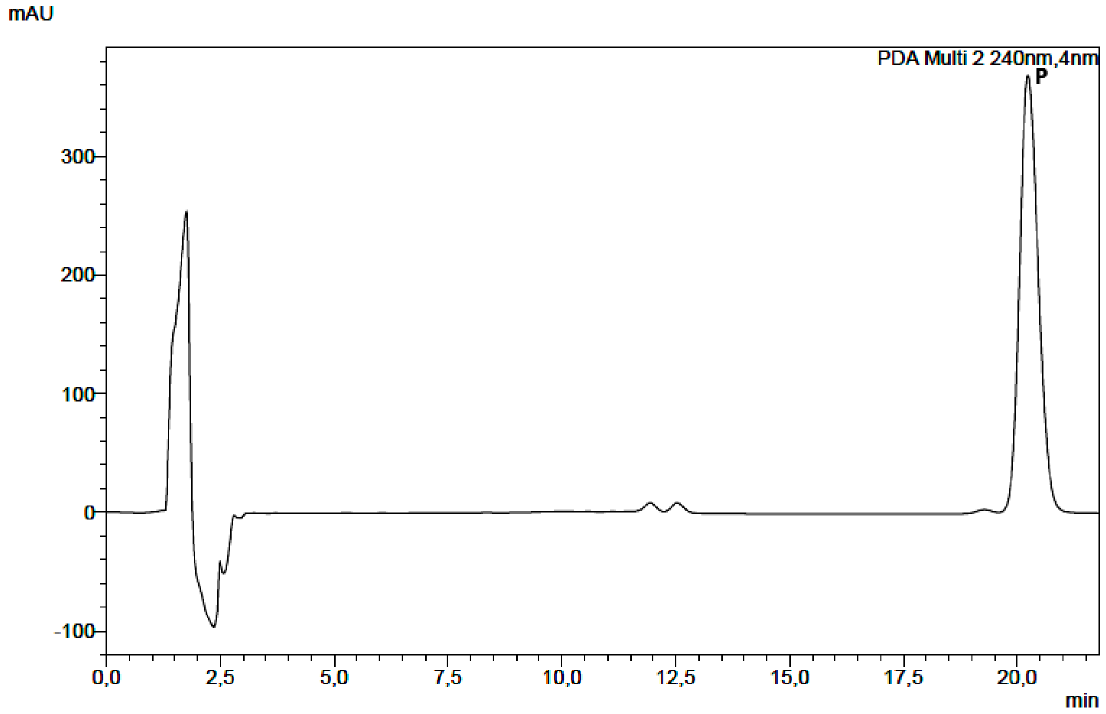

Figure S1F. Chromatogram obtained for palmatine on Polar RP column with mobile phase containing 28% MeCN, water and 0.04 ML^{-1} IL.

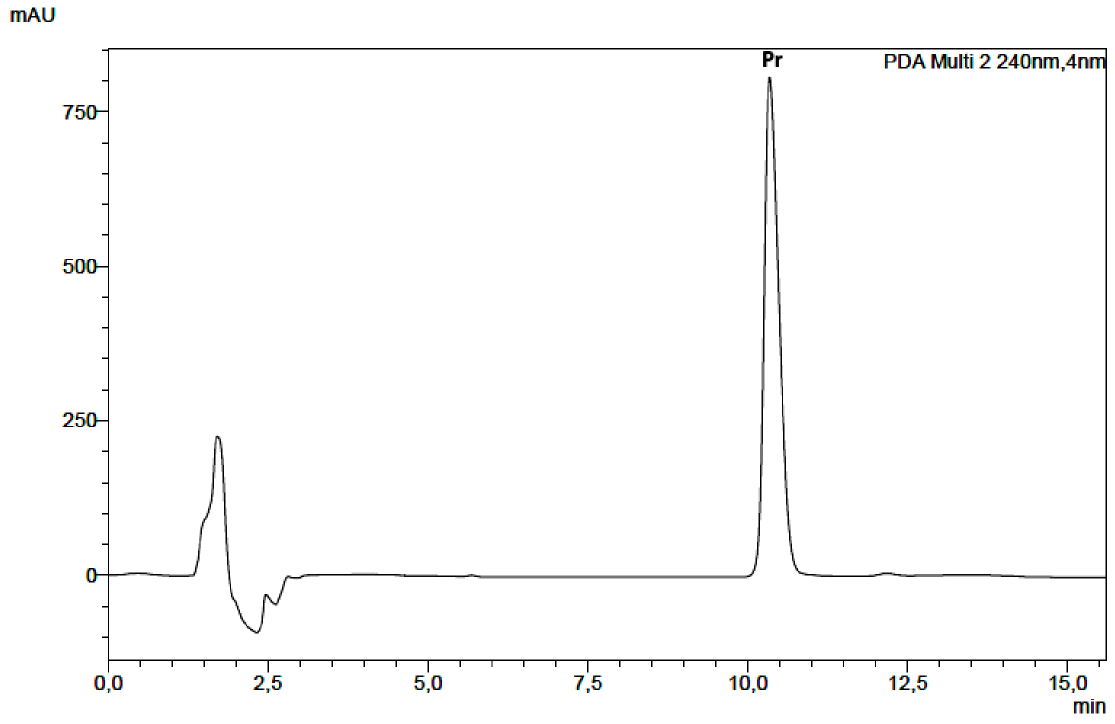

Figure S1G. Chromatogram obtained for protopine on Polar RP column with mobile phase containing 28% MeCN, water and 0.04 ML^{-1} IL.

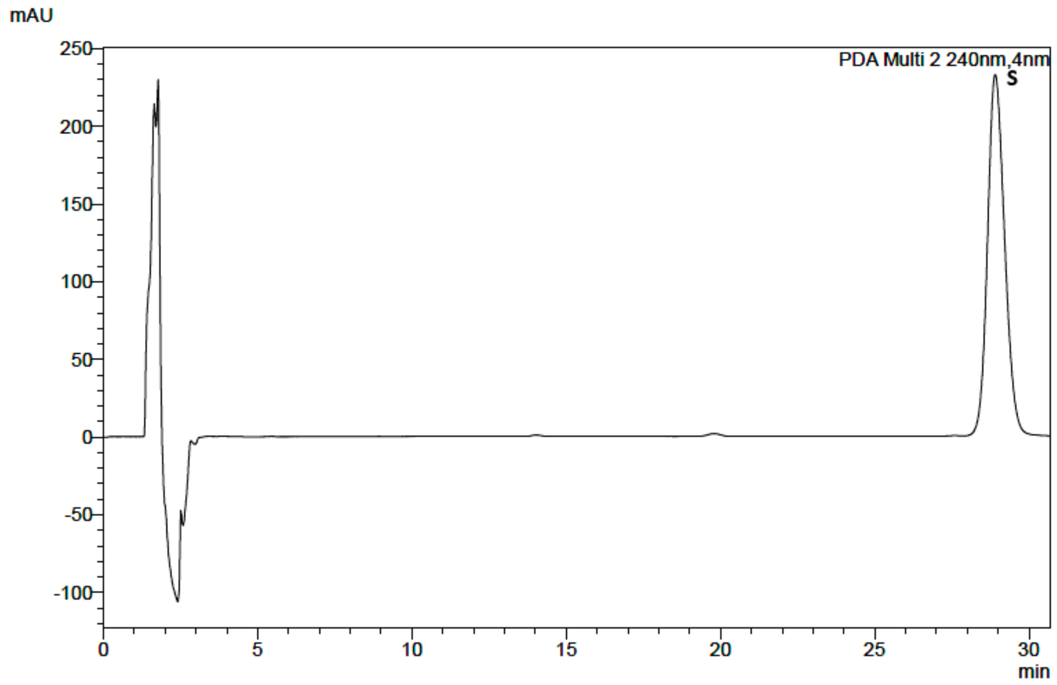

Figure S1H. Chromatogram obtained for sanguinarine on Polar RP column with mobile phase containing 28% MeCN, water and 0.04 ML^{-1} IL.

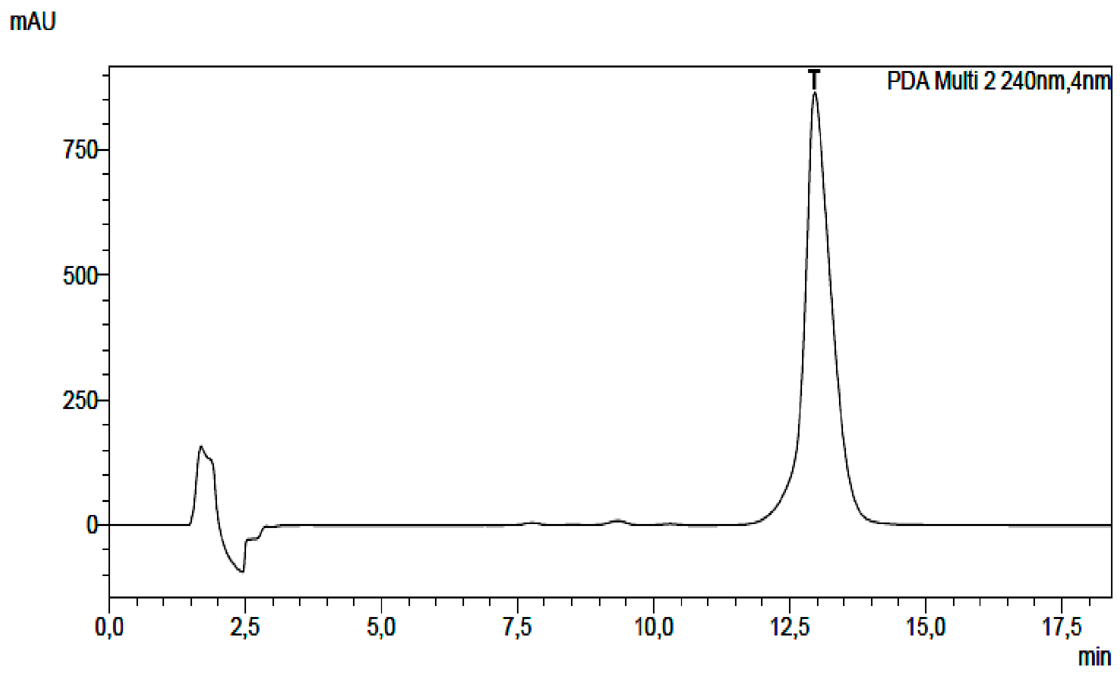

Figure S1I. Chromatogram obtained for tetrandrine on Polar RP column with mobile phase containing 28% MeCN, water and 0.04 ML^{-1} IL.

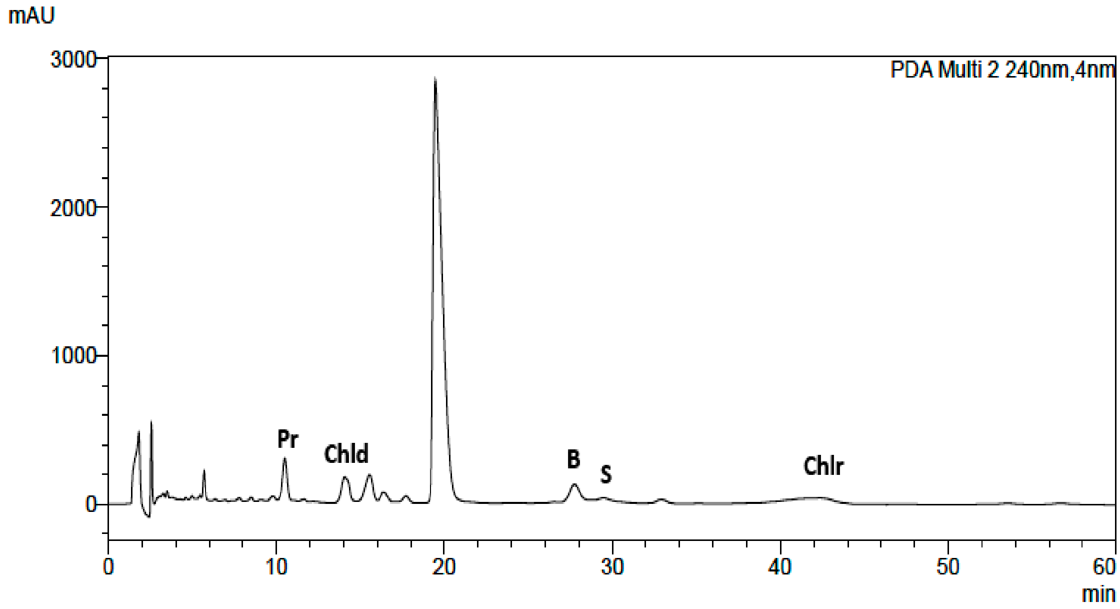

Figure S2. Chromatogram obtained for *Chelidonium majus* root extract obtained on Polar RP column with mobile phase containing 28% MeCN, water and 0.04 ML^{-1} IL. Abbreviations: Pr-protopine, Chld-chelidonine, B-berberine, S-sanquinarine, Chlr-chelerythrine.

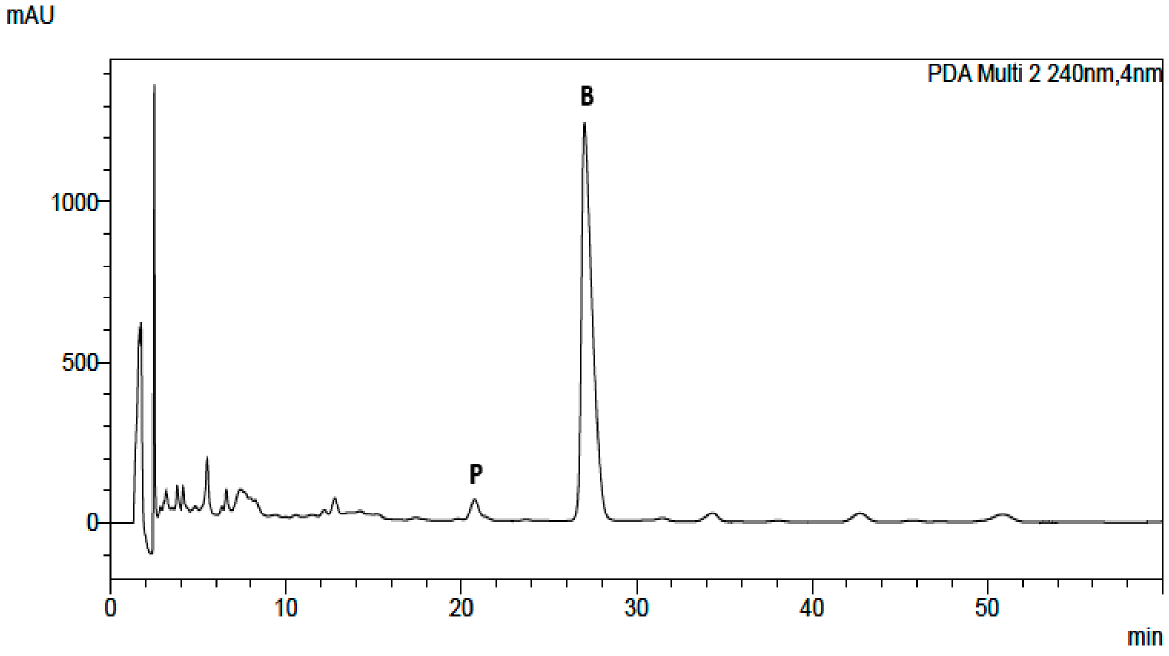

Figure S3. Chromatogram obtained for *Berberis vulgaris* cortex extract obtained on Polar RP column with mobile phase containing 28% MeCN, water and 0.04 ML^{-1} IL. Abbreviations: P-palmatine, B-berberine.

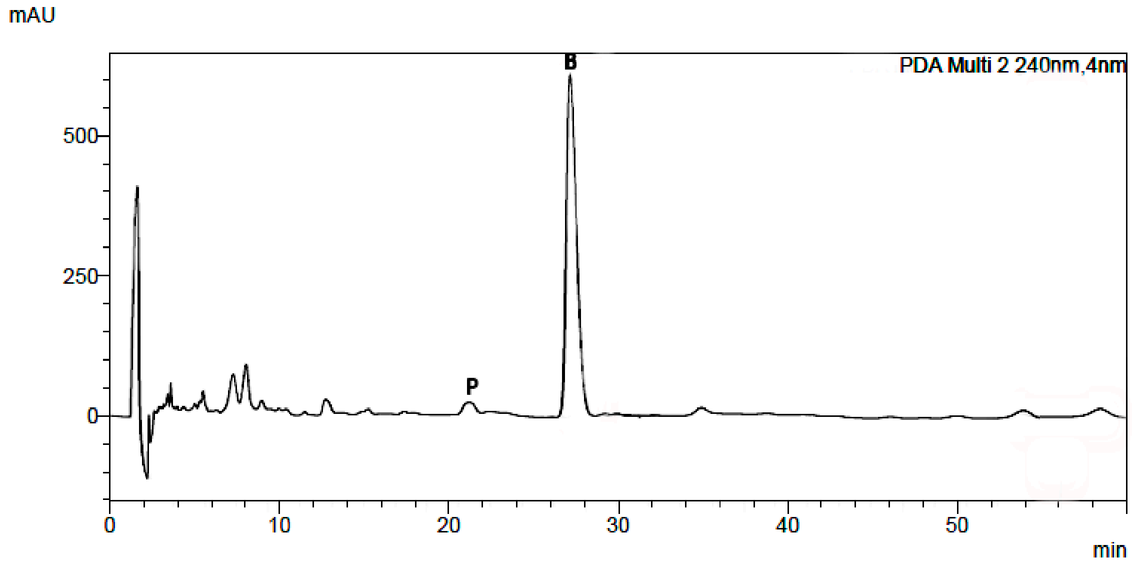

Figure S4. Chromatogram obtained for *Berberis thunbergii* cortex extract obtained on Polar RP column with mobile phase containing 28% MeCN, water and 0.04 ML^{-1} IL. Abbreviations: P-palmatine, B-berberine.

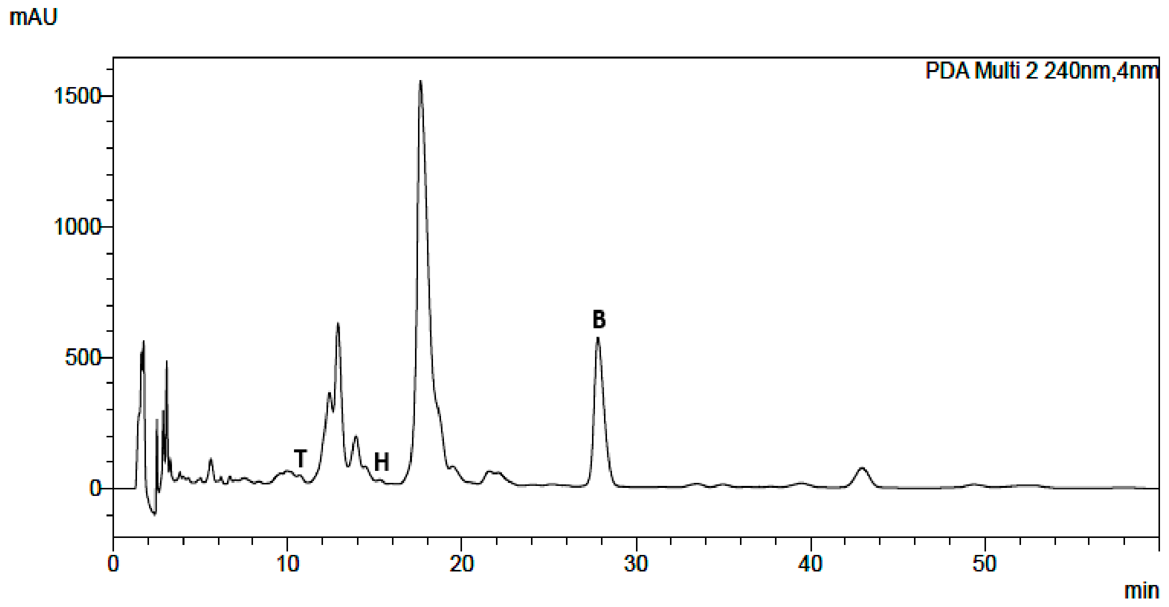

Figure S5. Chromatogram obtained for *Thalictrum foetidum* herb extract obtained on Polar RP column with mobile phase containing 28% MeCN, water and 0.04 ML^{-1} IL. Abbreviations: H-hernandezine, T-tetrandrine, B-berberine.
